# Radiographic and functional results in the treatment of early stages of Charcot neuroarthropathy with a walker boot and immediate weight bearing

**DOI:** 10.3402/dfa.v4i0.22487

**Published:** 2013-10-29

**Authors:** Maria Candida Ribeiro Parisi, Alexandre Leme Godoy-Santos, Rafael Trevisan Ortiz, Rafael Barban Sposeto, Marcos Hideyo Sakaki, Marcia Nery, Tulio Diniz Fernandes

**Affiliations:** 1 Division of Endocrinology, University of São Paulo, São Paulo, Brazil; 2 Department of Orthopedic Surgery, University of São Paulo, São Paulo, Brazil

**Keywords:** Charcot neuroarthropathy, classification, ulceration, diabetes, weight bearing

## Abstract

**Background:**

One of the most common gold standards for the treatment of Charcot neuroarthropathy (CN) in the early Eichenholtz stages I and II is immobilization with the total contact casting and lower limb offloading. However, the total amount of offloading is still debatable.

**Objectives:**

This study evaluates the clinical and radiographic findings in the treatment of early stages of CN (Eichenholtz stages I and II) with a walker boot and immediate total weight-bearing status.

**Methods:**

Twenty-two patients with type 2 diabetes mellitus (DM) and CN of Eichenholtz stages I and II were selected for non-operative treatment. All patients were educated about their condition, and full weight bearing was allowed as tolerated. Patients were monitored on a fortnightly basis in the earlier stages, with clinical examination, temperature measurement, and standardized weight-bearing radiographs. Their American Orthopedic Foot and Ankle Society (AOFAS) scores were determined before and after the treatment protocol.

**Results:**

No cutaneous ulcerations or infections were observed in the evaluated cases. The mean measured angles at the beginning and end of the study, although showing relative increase, did not present a statistically significant difference (*p >* 0.05). Mean AOFAS scores showed a statistically significant improvement by the end of the study (*p <* 0.005).

**Conclusion:**

The treatment of early stages of CN (Eichenholtz stages I and II) with emphasis on walker boot and immediate weight bearing has shown a good functional outcome, non-progressive deformity on radiographic assessment, and promising results as a safe treatment option.

Charcot neuroarthropathy (CN) is a chronic joint degeneration involving mainly the foot and ankle. It is associated with a myriad of conditions that cause loss of protective sensation in the lower extremities, such as tabes dorsalis, syringomyelia, Hansen's disease, congenital insensitivity to pain, and alcoholism. Today, diabetes mellitus (DM) is known to be the principal etiology. CN leads to progressive degeneration of affected joints and many authors consider it as the main complication of DM (1–4). Its prevalence in specialized services varies from 1 to 13%, and it is found to be 7.5% in patients with DM. It is bilateral in 10% of cases and in 60% the affected joint is the tarso-metatarsal, in 30% the Chopart's joint, and in 9% the tibiotarsal. Around 5% of the CN cases will have a recurrence, its characteristics are similar between both genders, and it is most commonly found in the first decade of DM (1, 3–6). The exact pathophysiology of CN joint degeneration remains under investigation, but present knowledge shows that sensory neuropathy allowing repeated microtraumas and autonomic neuropathy, associated with biological imbalance between osteoblasts and osteoclasts, lead to bone and joint destruction (6–9).

CN can be classified according to its clinical outcome and natural history. One of the most common utilized systems is the Eichenholtz (10) which divides the condition into three stages:

Eichenholtz I – stage of development, which is distinguished by clinical signs and symptoms of inflammation (warmth, erythema, and edema) and the visibility of radiographic changes. Common radiographic findings include bone debris formation at the articular margins, fragmentation of the subchondral bone, subluxation, dislocation, and capsular distention.

1. Eichenholtz II – stage of coalescence, is marked by decreased warmth, erythema, and edema. Radiographs show absorption of fine debris and fusion of large fragments to adjacent bones. The bone ends become sclerotic. At this point, the deformity ceases to progress and transitions to the reconstructive or remodeling stage.
2. Eichenholtz III – stage of reconstruction or remodeling, is characterized by rounding of the bone ends with a decrease in sclerosis, leading to consolidation. A structural bone deformity may be present and this resultant deformity may lead to skin breakdown and potential infection followed by amputation.

The usual treatment in Eichenholtz stage I and initial stage II is immobilization with the total contact casting and lower limb offloading. Immobilization should remain until the patient reaches Eichenholtz's stage III (healing). However, the time of offloading is debatable with some authors describing it up to 12 weeks (4, 11, 12). The main justification for this is that the affected foot is to be protected from further trauma exerted by the weight-bearing load, which would contribute to the destructive inflammatory process of the joint (4, 13–15). Surgical indications for CN reconstruction include structural deformities at risk for ulceration, significant instability, recurrent ulcerations, and localized infection (1, 16).

Studies of initial weight bearing in CN have been recently reported in the literature. Pinzur et al. (17) studied nine patients with diabetes and acute-phase CN, Eichenholtz stages I and II, using the total contact cast and weight bearing, with evaluation every 2 weeks, yielding promising results. At the end of the study, there was no anatomical difference between initial and final evaluations, and the subjects were able to use customized shoes. Using the same approach with longer follow-up, Souza, 2 years later, showed similar results (18).

As described, compliance with offloading prescription is poor (18), and the objective of this study was to evaluate the clinical and radiographic findings in the treatment of Eichenholtz stages I and II with the walker boot and immediate total weight bearing. In this study, we prescribed a walker boot – Robofoot (Salvapé Produtos Ortopédicos, São Paulo, Brazil) – for immobilization of patients with CN, instead of the total contact cast.

## Methods

After approval from the Scientific Commission University, 22 patients with type 2 DM and Eichenholtz stages I and II CN diagnosis were selected and submitted to the treatment protocol by the diabetic foot unit from January 2004 to January 2009. The inclusion and exclusion criteria were determined as shown below.

### Inclusion criteria

1. Patient with type 2 diabetes by the American Diabetes Association criteria (19);
2. CN Eichenholtz stages I and II without previous treatment;
3. Abnormalities in the neuropathy evaluation, performed with the 5.07/10 g Semmes-Weinstein monofilament (Sorry, Bauru, Brazil) and 128-Hz tuning fork (P. H. Industries Small Industries, Pakistan);
4. Endocrinological follow-up and glycemic control at the São Paulo University (São Paulo, Brazil);
5. Compliance with the proposed treatment protocol;
6. Regular follow-up with the institution's social services.

### Exclusion criteria

1. Presence of plantar foot ulcer at initial evaluation;
2. Preceding surgical procedure on affected foot;
3. Preceding osteomyelitis;
4. Presence of rheumatological and immunological diseases or alcoholism;
5. Patient on hemodialysis;
6. Contralateral limb amputation;
7. Pregnancy;
8. Cognitive impairment that would hinder comprehension of orientations and medical prescriptions.

## Treatment protocol

After clinical and radiographic diagnosis, based on the criteria described in Table 1, all patients agreed with the treatment conditions without any other medical imaging studies and their American Orthopedic Foot and Ankle Society (AOFAS) (20) scores were determined. The patients received a walker boot – Robofoot^®^ – on the day of initial evaluation, as well as instructions for the adequate utilization of the equipment. Specific orientations about the clinical situation of CN, risks and implications, and compliance necessity, were given, as well as prescription reinforcement. At the same time, patients were told to bear weight respecting symptomatic limitations of each case.

**Table 1:** Clinical and radiographic parameters evaluated

Vascular	Neuropathy	Osteoarticular	Cutaneous	Radiographic
Pulses (tibial and pedal)	Pain	Equinus of the foot	Ulcer	Joint congruence
Hyperemia	Proprioception	Clawed toes	Hyperkeratosis	Bone destruction
Edema	Dehydration	Instability	Infection	Talar-first metatarsal angle
Comparative temperature				Flatfoot

Subjects were monitored every 15 days during the first 12 weeks and monthly thereafter. In all evaluations, a thorough clinical examination was performed, including local temperature, skin abnormalities, and standardized radiographic evaluations. The walker boot was discontinued when patients had shown all three parameters (clinical, radiographic, and temperature measurement of comparative lower limbs) as described below:

1. Clinical: no pain, warmth, erythema, or edema.
2. Temperature: when the temperature difference between lower extremities had dropped to less than 2**°**C (21).
3. Radiographic: bones with a decrease in sclerosis and signals of consolidation.

The mean time of treatment of the studied population was 18 weeks. Anteroposterior and lateral foot radiographs in weight-bearing position were performed at the beginning of the treatment, at 6 weeks, and at the end of treatment. The radiographic evaluation of the osseous anatomic pattern and progression, as well as the forefoot abduction and decrease of the medial longitudinal arch was made through the measurement of the angle between the talus and first metatarsal on the lateral radiograph using a simple goniometer. Its assessment was performed by two different evaluators. The agreements between the intraobserver and interobserver were demonstrated high for images evaluated (*κ* 0.984/*κ* 0.981).

**Fig. 1:**
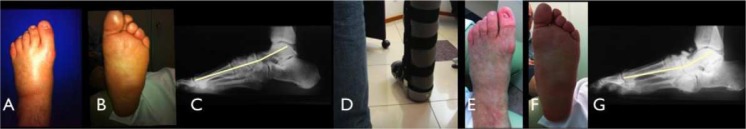
Clinical and radiographic views of left foot of patient number 5, at the beginning (A, B, C) and end of treatment (D–G). (A) Dorsal foot view, (B) plantar foot view, (C) weight-bearing foot lateral radiograph, (D) walker boot, (E) dorsal foot view, (F) plantar foot view and (G) weight-bearing foot lateral radiograph.

The clinical evolution of the foot and ankle was studied by using the American Orthopedic Foot and Ankle Society Ankle-Hindfoot Scale (20) which was applied at the beginning and end of the study. All patients studied were under clinical follow-up until the end of this study (Fig. 1).

Statistical analysis of the results was made by using the Statistical Analysis System (SAS Institute Inc., 1985) and Student's *t*-test was used to compare parametrical data. The significance level was settled at *p <* 0.05.

## Results

All patients from the studied data had type 2 diabetes; 7 were males and 15 were females, mean age was 56 years (47–64), mean time since DM diagnosis was 13 years (8–25), and mean body mass index was 28 (23–34) as shown in Table 2.

**Table 2:** Clinical and demographic values

Patients	Gender	Age	Time of diabetes (years)	Body index mass
1	F	54	15	28
2	F	56	20	31
3	F	62	14	26
4	M	64	18	25
5	F	50	12	27
6	F	48	18	24
7	M	47	14	26
8	F	62	18	27
9	F	60	16	29
10	F	50	25	34
11	F	60	9	23
12	M	49	8	31
13	M	51	14	26
14	F	52	9	28
15	F	54	10	33
16	F	50	15	27
17	F	64	12	25
18	M	61	8	34
19	M	61	11	31
20	M	64	12	28
21	F	53	8	28
22	F	62	10	26

F, female; M, male.

Vascular characteristics such as hyperemia, edema, and comparative temperature, showed improvement by the end of treatment and there was no change in the pulse pattern. Cutaneous dehydration resolved when positive at the beginning of treatment and clawing of the digital deformities was unaltered until the end of the study. Cutaneous ulcerations and infections were not observed in the evaluated cases. There was hyperkeratosis improvement in all patients. Clinical characteristics and mean AOFAS score are described in Table 3. Mean AOFAS scores showed a statistically significant improvement by the end of the study (*p <* 0.005). The mean measured talar-first metatarsal angle at the beginning and end of the study, although showing relative increase, did not present a statistically significant difference (*p >* 0.05) as shown in Table 4.

**Table 3:** Clinical findings and mean AOFAS score

	Beginning of treatment	End of treatment
Pulses (tibial and pedal)	Present	Present
Hyperemia	20 present/2 absent	22 absent
Edema	22 moderate or severe	22 mild
Comparative temperature	4° (mean)	1° (mean)
Pain	22 present	22 absent
Dehydration	15 + /7 absent	22 absent
Clawing of fingers	17 + /5 absent	17 + /5 absent
Instability	3 + /19 absent	22 absent
Flatfoot	16 + /6 absent	22 present
Forefoot abduction	14 + /8 absent	22 present
Ulcer	22 absent	22 absent
Hyperkeratosis	16 + /8 absent	22 absent
Infection	22 absent	22 absent
AOFAS	40,54	75,04

AOFAS, American Orthopedic Foot and Ankle Society.

**Table 4:** Angle values and AOFAS score

	Beginning of treatment		End of treatment	
		
Patients	Initial angle	AOFAS score	Final angle	AOFAS score
1	1	40	1	80
2	10	41	10	74
3	7	40	8	72
4	8	39	9	76
5	15	38	17	74
6	15	40	16	73
7	10	38	12	75
8	9	40	10	75
9	9	41	9	74
10	11	40	11	76
11	10	42	10	70
12	11	40	11	72
13	9	41	9	76
14	6	44	6	79
15	6	39	7	77
16	3	43	4	78
17	6	40	7	77
18	7	42	8	78
19	9	39	10	73
20	9	41	9	74
21	6	44	5	70
22	7	40	7	78

AOFAS, American Orthopedic Foot and Ankle Society.

## Discussion

The pathophysiology of CN still remains under investigation and the best treatment option is still a matter of debate. The clinical characteristics of our population were quite similar to previous studies regarding not only the affected joint anatomy (tarso-metatarsal joint) but also the age and time since diagnosis (22–24). Many studies recommended the use of total contact casting and no weight bearing on the affected CN limb as the initial therapy of choice to avoid cutaneous complications, joint instability, and large bone deformities, and also provide symptomatic relief (13, 25–29). However, practical difficulties in limb offloading and non-compliance were identified in some studies and also in this study which were mainly attributed to patient adaptation to the use of orthosis, diminished proprioception and equilibrium, locomotion difficulties, need to maintain labor activities, and large number of medical appointments (18, 27–29).

Studies (17, 18) designed to assess treatment safety in these patients, when involving precocious weight bearing in Eichenholtz stages I and II, showed low rates of complications, which encouraged the start of the non-surgical, aggressive treatment protocol for diabetic patients in our institution. Despite our limited selected group, precise parameters of follow-up, and treatment protocol safety; limitations of the present study could best be addressed by a research case series design that could have affected the internal validity and generalization of clinical outcomes.

The multidisciplinary team approach in our institution, systematic patient follow-up, emergency medical assistance, and active search for complications of the diabetic foot syndrome allowed us to rapidly identify the clinical presentation of the disease and promptly institute the proposed treatment. The clinical characteristics elected for evaluation in this study presented stability or a trend toward improvement at the end of the study that was found similarly in other studies (17, 18).

The most worrisome and prevalent complications, such as ulcerations and infections, were not seen in the studied group; data that were reported in previous studies (17, 18). The flatfoot and abduction deformities affected all subjects by the end of treatment, although radiographic angle progression that represents them did not show statistical difference. This biomechanical behavior represents the healing phase of CN and does not necessarily indicate worsening of these patients’ prognosis. The AOFAS score progress was not only statistically significant, but also encouraging, reflecting the symptomatic and functional improvement of all patients subjected to this approach.

## Conclusion

The treatment of early stages of CN (Eichenholtz stages I and II) with emphasis on walker boot and immediate weight bearing has shown a good functional outcome and non-progressive deformity on radiographic assessment and may therefore be a safe treatment option.
